# Gut microbiota dysbiosis induces neuroinflammation in major depressive disorders: mechanisms targeting the gut-brain axis

**DOI:** 10.3389/fpsyt.2025.1629182

**Published:** 2025-09-18

**Authors:** Jiayi Li, Bei Wan, Le Zhou, Xin Qian, Fushun Wang, Simeng Gu, Xianjun Ma, Jason H. Huang

**Affiliations:** ^1^ Department of Psychology, Jiangsu University Medical School, Zhenjiang, Jiangsu, China; ^2^ Department of Psychology, Sichuan Normal University, Chengdu, Sichuan, China; ^3^ Department of Neurology, Nanjing University of Chinese Medicine Affiliated Hospital of Lianyungang (Lianyungang Hospital of Traditional Medicine), Lianyungang, China; ^4^ Department of Neurosurgery, Baylor Scott and White Health Center, Temple, TX, United States; ^5^ Department of Surgery, Texas A and M University, Temple, TX, United States

**Keywords:** neuroinflammation, MDD, gut-brain axis, microglia, astrocytes

## Abstract

Major depressive disorder (MDD) is a kind of mental disorder with high mortality, suicide and relapse rates, and might be the world's leading cause of health burden by 2030. Growing evidence suggests that neuroinflammation is closely linked to depressive pathogenesis and suggests that MDD can be called a microglia disease. And activation of the P2X7R/NLRP3 signaling pathway in microglia is a key mechanism causing nerve damage. In addition, it is recently found that gut microbiota might initiate neuroinflammatory processes underlying MDD, and gut microbiota dysbiosis can be affected by sleep to ameliorate neuroinflammatory processes. In this paper, we reviewed recent advances about gut-brain axis interactions with neuroinflammation, which might shed light on the mechanisms and treatment of depression.

## 1 Introduction

Major Depressive Disorders (MDD) is manifested primarily as enduring depressed mood, psychomotion retardation, cognitive impairment, with some somatic symptoms such as sleep disturbances, fatigue, and appetite changes (e.g., weight loss in most cases, though increased appetite and weight gain may occur in some patients), as well as loss of libido. Global statistics have indicated approximately half of annual 800,000 suicide deaths are due to individuals diagnosed with MDD. Notably, depressed individuals exhibit a nearly 20-fold higher risk of suicide mortality compared with the general population (1). The World Health Organization (WHO) predicts that MDD will become the leading cause of global disease burden by 2030 (2). Even though the incidence rate of MDD is high, the precise etiology and pathogenesis of MDD remain elusive.

The monoamine hypothesis has dominated MDD research, and the first-line treatment for MDD are still conventional antidepressants which primarily target monoamine neurotransmitter modulation (3–5). However, these conventional medications demonstrate limited efficacy, with only 30-50% of the cases having been fully recovered, but frequently causing adverse effects such as sexual dysfunction and weight gain (6). It is important to elucidate novel pathogenic mechanisms and identify alternative treatment targets. Recent studies has been increasingly focused on the immune-inflammatory hypothesis, possibly due to the high comorbidity rates between MDD and chronic inflammatory conditions such as rheumatoid arthritis, cardiovascular disease, metabolic syndrome, and inflammatory bowel disease (7), which points to potential pathophysiological mechanisms.

In addition, emerging evidence highlights the critical role of the gut-brain axis (GBA) in neuropsychiatric disorders, including MDD, anxiety, and Alzheimer’s disease. Promisingly, the probiotic *Bifidobacterium adolescentis* has demonstrated therapeutic effects by reducing hippocampal pro-inflammatory cytokine levels (e.g., IL-1β, TNF-α) and ameliorating depression-like behaviors in chronically stressed mouse models (8), suggesting microbiota modulation might be a possible treatment strategy. Gut microbiota dysbiosis has been shown to induce neuroinflammation in the ventral hippocampus, a key brain region implicated in mood and cognition functions of brain (9). However, most of the tradition studies only discussed the effects of gut microbiota on MDD or neuroinflammation on MDD. Some recent studies found that gut microbiota might affect MDD via modulating neuroinflammation (10, 11), and based on this viewpoint, this paper aims to critically examine recent evidence on the effects of gut microbiota that influence the neuroinflammation and thus MDD.

## 2 Gut-brain axis

The brain regulates gastrointestinal sensory, motor, and secretory functions via neuroendocrine pathways including the hypothalamic-pituitary-adrenal (HPA) axis and autonomic nervous system. The gut in turn influences the central nervous system (CNS) through different pathways such as gut microbiota-derived metabolites, neurotransmitters, hormones, etc., which influence the enteric nervous system (ENS) and the immune system. This complex bidirectional pathway can be called the brain-gut axis (GBA). The gut microbiota (GM), comprising over 10^14^ microorganisms across four dominant phyla (*Bacteroidetes, Firmicutes, Actinobacteria, Proteobacteria*), plays a vital role in GBA dynamics. While maintaining relative stability throughout adulthood, microbial composition demonstrates plasticity in response to physiological dysfunctions and psychological stress. The changes of gut microbiota not only leads to increased local inflammatory responses within the gut but also induces neuro- inflammation in the brain, especially in the stress-related brain regions (12).

Many substances are involved in the gut-brain axis, such as carbohydrates, proteins, and peptides that are not absorbed by the host. These substances can be metabolized by intestinal microorganisms to produce a variety of biologically active substances, including amino acids, short-chain fatty acids (SCFAs), organic acids, phenolic compounds, phenylalanine derivatives, and indoles (12). These microbial metabolites might serve two functions: as major energy substrates for the colonic epithelium and as key signaling molecules for gut-brain communication. During stressful situations, such as gut microbiota dysbiosis, the intestinal barrier integrity can be impaired via structural and functional alterations. This barrier dysfunction facilitates pathogens and pro-inflammatory molecules into systemic circulation to initiate oxidative stress cascades and neuroinflammation in the brain. By integrating neuroimmune and endocrine pathways, they link gut microbial ecology to brain inflammatory states and mood regulation dynamics.

### 2.1 Gut-brain axis and sleep

Emerging research showed that sleep disorder is not only an important process that accompany MDD, but also showed significant cross-talk with the gut microbiota. Many recent studies have proved that microbiota can affect sleep through some specific pathways, and gut microbiota might be the reason for sleep changes at ageing, thus both the gut microbiota and sleep changes are suggested to be significant signatures that are associated with health and disease in the last decades of life (13). A number of studies have shown that the composition of the gut microbiota undergoes significant changes with age (14, 15); and it was shown that general age-related changes in the intestinal microflora include the reduced number of species and quantitative composition of Bifidobacteria in the elderly people is the decrease in (16). One of the possible explanations for their adhesion to the intestinal wall due to changes in the chemical composition and structure of the colon mucous membrane. The changes in the bacteria, in turn, can affect the permeability of the intestinal wall, and also the release of some neurotransmitters, such as aminobutyric acid (γ-Aminobutyric acid, GABA) (17, 18) and serotonin, which are important neurotransmitters that is involved in sleep neurotransmitter and metabolic processes in the brain. Very interestingly, it is found that the bacteria *Bifidobacterium* and *Lactobacillus* are actively involved in the production of aminobutyric acid (γ-Aminobutyric acid, GABA) (17, 18).

In addition, it is of our interest that chronic sleep disruption impacts gut microbiota. A study in animals has revealed that chronic sleep fragmentation alters taxonomic profiles of fecal microbiota and induces systemic and adipose tissue inflammation and insulin resistance (19). In another research, better sleep quality in healthy older adults was associated with better neuropsychological test performance and higher abundance of microbial *phyla Verrucomicrobia* and *Lentisphaerae* in the stool samples (20). In addition, many studies have confirmed the causal relationship between gut microbiota and sleep disorders, and some studies really found that changes in gut microbiota composition might be the reasons for age-related changes in sleep. Furthermore many sleep disorders are due to instable gut microbiota, and changes with dietary composition can achieve sound sleep via maintaining microbiota homeostasis (21). Thus microbiota-targeted interventions might be a good way to improve sleep, and sleep in turn can affect microbiota composition to improve emotional disorders, particularly for MDD patients experiencing high stress or with sleep complaints (22).

### 2.2 Gut-brain axis and neuroinflammation

Neuroinflammation is an inflammatory response caused by dysregulation of the synthesis and release of various pro-inflammatory and anti-inflammatory cytokines in the brain (23). The inflammatory response may be caused by infection, autoimmune disease, trauma, or other factors. Recent studies found that dysbiosis of intestinal flora is an important factor for neuroinflammation by changing the inflammatory factors. For example, it is found that disruption of the gut microbiota reduces short chain fatty acids (SCFAs) levels, hindering their anti-inflammatory effects. Dysbiosis of intestinal flora can damage the intestinal barrier, allowing pathogen-associated molecular patterns (PAMPs) such as bacterial lipopolysaccharides (LPS) to activate innate immune cells (e.g., macrophages, dendritic cells) and Toll-like receptors (e.g., TLR4) on intestinal epithelial cells, triggering the NF-κb pathway and releasing pro-inflammatory factors and chemokines. This further leads to the transmission of immune stimulation signals from the periphery to the central nervous system, resulting in an increase in Th17 cells (pro-inflammatory) and a decrease in Treg cells (anti-inflammatory). Peripheral monocytes can migrate into the brain, and differentiate into M1-type macrophages to release high levels of IL-1β, IL-6, TNF-α, etc., which in turn disrupt the integrity of the blood-brain barrier (BBB) and induce neuroinflammation (24).

Clinical studies have discovered the presence of various inflammatory mediators such as IL-1β, IL-6, tumor necrosis factor α (TNF-α), Toll-like receptor 3 (TLR3), and Toll-like receptor 4 (TLR4) in brains of patients with MDD patients who committed suicide, and the levels of these inflammatory factors were significantly higher than those of normal individuals (25). And it is suggested that activation of microglia and astrocytes is a characteristic change in neuroinflammation, and their activation increases the expression of pro-inflammatory cytokines and/or the production of reactive oxygen species (ROS), which results in neuronal damage, characterized by diminished neurogenesis, reduced dendritic spine density, and impaired synaptic plasticity (26–28). These alterations might affect mood and cognitive function, thereby increasing the risk of MDD (26).

### 2.3 The GBA and neuroinfllamation in MDD

In recent years, with the rapid advancement of molecular biology, the gut microbiota has become a prominent topic in scientific research. Gut microbiota are composed of several species of microorganisms, including bacteria, yeast, and viruses. The dominant gut microbial phyla are *Firmicutes, Bacteroidetes, Actinobacteria, Proteobacteria, Fusobacteria*, and *Verrucomicrobia*, with the two phyla *Firmicutes* and *Bacteroidetes* representing 90% of gut microbiota. The *Firmicutes* phylum is composed of more than 200 different genera such as *Lactobacillus*, *Bacillus*, *Clostridium*, *Enterococcus*, and *Ruminicoccus*. *Clostridium* genera represent 95% of the *Firmicutes* phyla. *Bacteroidetes* consists of predominant genera such as *Bacteroides* and *Prevotella*. The *Actinobacteria* phylum is proportionally less abundant and mainly represented by the *Bifidobacterium* genus (29). Gut microbiota composition is highly variable and the variation itself is considered as physiological in the context of healthy gut microbiota, according to age, ethnicity, lifestyle, and dietary habits. However, these physiological gut microbiota variations have huge implications in intestinal and extra-intestinal disorders. Indeed, dysbiosis is often defined as an alteration of gut microbiota composition and a cause or a consequence of disorders. It is often difficult to ascertain whether the change is beneficial or detrimental (30), even though *Lactobacillus reuteri*, *Blautia producta*, *Lactobacillus* and *Bifidobacterium* strains shows beneficial effects.

Dysbiosis of the gut microbiota has been increasingly linked to a variety of chronic diseases, including metabolic, immune, and neurological disorders (30). Animal studies found that rodents exposed to the chronic unpredictable mild stimulus (CUMS) do undergo significant changes in gut microbiota structure, manifested as significant increase in the relative abundance of *Odoribacter, Rikenella, Streptococcus, Anaerotruncus*, *Clostridium, and Helicobacter* (31). Parallel findings in chronic social defeat stress (CSDS) models reveal simultaneous emergence of depressive-like behaviors and gut dysbiosis (32). The causal role of gut microbiota in MDD pathophysiology is further substantiated by fecal microbiota transplantation (FMT) studies. Transplantation of MDD patient-derived microbiota into germ-free mice recapitulates core behavioral deficits, including anhedonia, locomotor hypoactivity, and weight loss, with behavioral severity correlating strongly with systemic IL-6 levels (33). Paradoxically, transplantation of healthy individuals microbiota into germ-free rodents induces transient depressive-like states, suggesting bidirectional microbiota-brain communication may involve neuroimmune priming mechanisms.

In addition, antibiotics can clear the gut of bacteria, thereby reducing bacterial translocation, decreasing the liver burden, and alleviating neuroinflammation, thus providing therapeutic effects. For example, rifaximin, an unabsorbable, broad-spectrum, gastrointestinal-specific antibiotic, lowers serum ammonia and endotoxin levels, potentially alleviating symptoms (34). Antibiotic administration attenuated behavioral impairments, mitigated gut dysbiosis, reduced intestinal inflammation, and partially rescued MDD. The results highlight the antibiotic treatment as a potential therapy for opioid withdrawal sequelae (35). In addition to combating dysbiosis, certain antibiotics, such as minocycline and doxycycline, have been shown to inhibit matrix metalloproteinase activity and prevent mitochondrial dysfunction, microglial activation, offering therapeutic benefits (36). Another study has demonstrated that Prebiotics specifically Fructooligosaccharides (FOS) and Galactooligosaccharides (GOS) can affect neuroinflammation, depression, and anxiety-like behavior in a mouse model fed a high-fat diet (HFD) (37). Despite these beneficial effects, the use of antibiotics remains controversial, with evidence indicating that long-term use can eliminate beneficial bacteria, leading to dysbiosis and disease exacerbation.

Compared with healthy populations, patients with MDD exhibit significant alterations in gut microbiota diversity and taxonomic composition. Metagenomic analyses found that MDD patients demonstrate elevated relative abundances of *Eggerthella(Actinobacteria), Subdoligranulum*, and *Coprococcus (Lachnospiraceae)*, alongside marked reductions in SCFA-producing bacteria such as *Ruminococcaceae.* This dysbiosis is particularly pronounced in the inflammatory MDD subtype, with an increase in Bacteroidetes phylum abundance and a decrease in *Clostridiales* order abundance. Notably, the *Sellimonas* genus shows dose-dependent positive correlation with MDD severity, exhibiting pro-inflammatory effects potentially mediated via the TLR4/NF-κB pathway (38). Conversely, change in the bacteria can shape local and systemic immune responses, and it is found that microbial metabolites, including short-chain and branched-chain fatty acids, bile acids, tryptophan derivatives, and others, influence local and systematic immune cells (39, 40), including resident activated T lymphocyte, including IL-10 producing, T-bet expressing CD4+ Tr1 T cells (41).

GBA-targeted interventions has demonstrated potential antidepressant therapy effects, and it is found that matrine can reverse CUMS-induced Firmicutes/Bacteroidetes ratio imbalance in mice, concurrently upregulating hippocampal BDNF expression to ameliorate depressive behaviors (42). Similar study in Wistar-Kyoto depressive model rats, found that 1-month *Lactobacillus helveticus* NS8 intervention increased fecal bacterial abundance to 8.7-fold and reversed anhedonia (43). In contrast, chronic stress induced MDD in mice, and led to significant reduction in indole-3-lactic acid (ILA) in both the gut and brain, and supplementation with the psychobiotic Bifidobacterium breve CCFM1025 (Bre1025) restored ILA concentrations to normal levels (44). In addition, traditional Chinese formula Jiannao Wan alleviates chronic restraint stress (CRS)-induced anxiety-like behaviors through Akkermansia muciniphila enrichment and modulation of tryptophan-serotonin metabolism (45). Furthermore, gut-selective antibiotic rifaximin prevents CUMS-induced firmicutes reduction and suppresses hippocampal IL-1β overexpression, with the therapeutic efficacy correlating with restored microbiota-derived SCFA levels (27).

**Figure 1:**
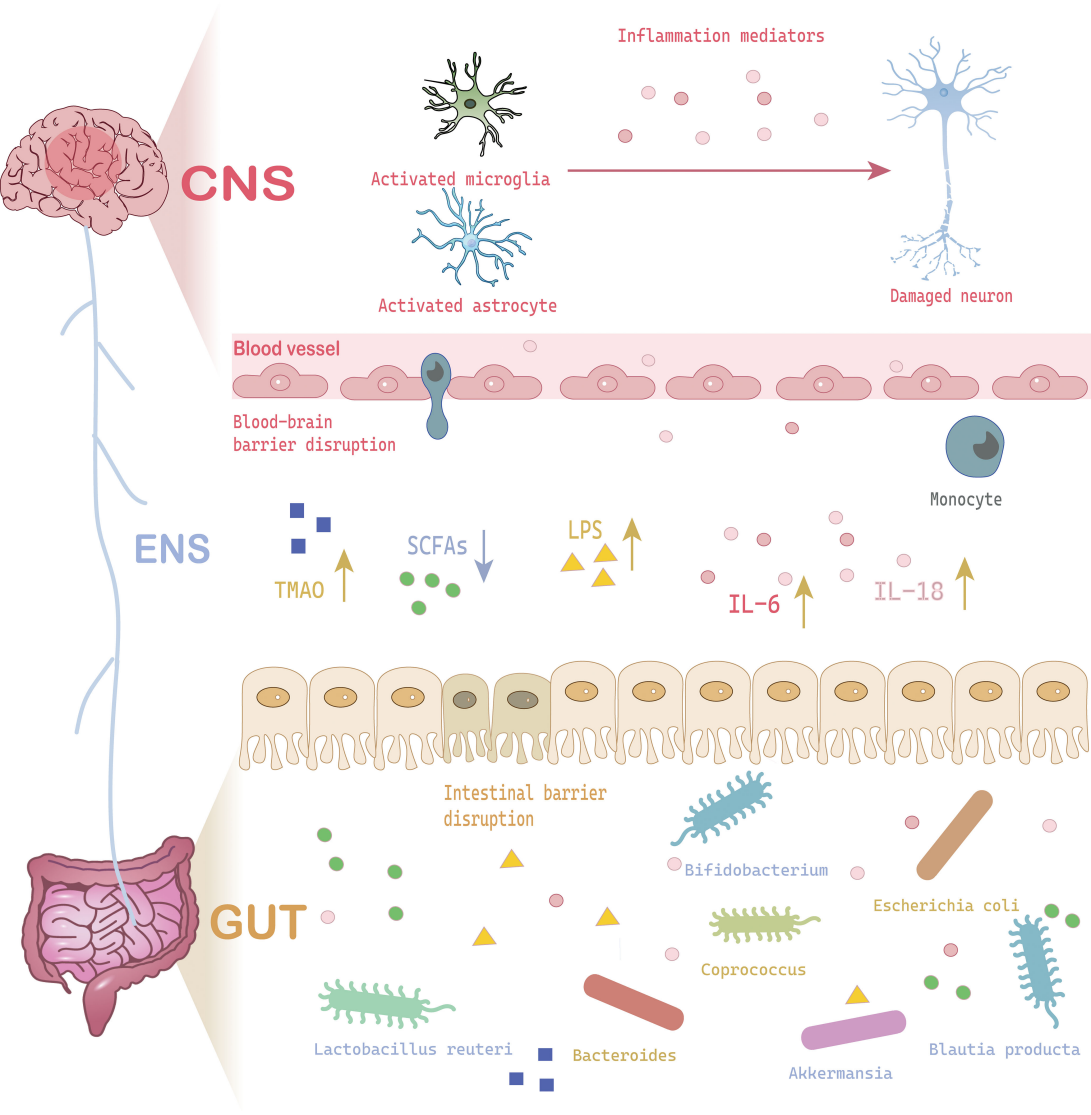
A schematic cartoon shows the interaction between gut microbiota and neuroinflammation to induce MDD. The brain-gut-microbiota axis interacts through neural signaling, immune signaling, and chemical signaling. When gut microbiota dysbiosis occurs due to stress or other factors, the released metabolites (SCFAs, LPS, TMAO) can cause damage to the CNS (increased BBB permeability, glial cell activation, neuronal damage). The stress response to CNS damage can further alter the gut microbiota, stimulating inflammatory responses, creating a vicious cycle that leads to MDD. CNS, Central Nervous System; IL-6, Interleukin 6; IL-18, Interleukin 18; LPS, Lipopolysaccharide; SCFA, Short-Chain Fatty Acids; TMAO, Trimethylamine N-Oxide.

Recent studies have found that gut microbiota-derived metabolites play a pivotal regulatory role in the pathogenesis of MDD (**Figure 1**) (12). SCFAs significantly improve brain function through multiple pathways, including modulating blood brain barrier (BBB) permeability, suppressing neuroinflammatory responses, and promoting hippocampal neurogenesis. Notably, butyrate, acting as a histone deacetylase (HDAC) inhibitor, exerts neuroprotective effects via epigenetic regulatory mechanisms. Importantly, alterations in gut microbiota composition can simultaneously influence both SCFAs biosynthesis and neurotransmitter production (e.g.5-HT), and this dual regulatory mechanism holds significant implications in depressive pathology. Wu et al. (46) demonstrated that depressive mice exhibited significantly reduced abundance of *Akkermansia* genus, which showed strong positive correlations with acetate and 5-HT levels (46). Emerging research has unveiled interactions between environmental factors and gut metabolites: Ethanol exposure can induce intestinal structural/functional abnormalities and trigger neuroinflammation, ultimately leading to depressive-like behaviors, while exogenous supplementation of SCFAs can effectively reverse these pathological alterations (47). Similarly adenosine supplementation increased serum SCFA levels in mice, thereby alleviating MDD-like behaviors in CSDS mice (48). Beyond protective metabolites, certain microbiota-derived metabolites may exhibit neurotoxicity, for example, lipopolysaccharide (LPS) not only inhibits hippocampal neural progenitor cell (NPCs), it can also induce neuronal apoptosis through activation of the TLR4 signaling pathway, which is a pro-inflammatory process particularly prominent in neurodegenerative diseases. Similarly, quinovic acid glycosides derived from gut microbiota have been proposed to be activators of neuroinflammatory pathways. These gut microbiota-derived compounds might work as activators for brain-resident immune cells (e.g., microglia) and the release of pro-inflammatory cytokines, which may induce cytotoxicity toward neurons (38, 49). Gut microbiota such as *Bacteroidetes* and *Firmicutes* phyla might participate in producing precursor molecules of trimethylamine N-oxide (TMAO), which has been closely linked to neurological disorders, including MDD (50). Collectively, these findings shed light on the effects of gut microbiota, which might play a pivotal role in the pathogenesis of MDD through direct or indirect mechanisms (**Figure 1**).

## 3 Core pathways of gut microbiota in regulating neuroinflammation

### 3.1 Gut microbiota-glia axis in neuroinflammation

The results of a growing number of studies (51, 52) now support the presence of the characteristic alterations of neuroinflammation in MDD, which are mainly manifested as microglia activation and/or accompanied by astrocyte activation, etc (**Figure 1**). The gut microbiota and glial cells play an important interplay in driving neuroinflammation that leads to MDD. Microglia (MG) are resident immune cells of the CNS, working as resting and activated states. Microglia activation is commonly seen in response to injury, ischemia, and other stimuli. Activated microglia showed enhanced phagocytosis and can secrete various inflammatory factors, complement system, ROS, and other mediators (53). Based on their phenotypic expression, activated microglia can be categorized into two distinct polarization states, M1 and M2, which can be further identified by recognizing markers and morphology under different conditions. The morphological changes of microglia are diverse, roughly including six types: Ramified type, Amoeboid type, Bulbous endings of microglial processes type, Ball-and-chain structures type, Hyper-ramified type, Jellyfish type, and Rod type (54). Commonly used microglial markers for the M1 phenotype include MHC-II, CD16, CD32, CD80, CD86, CD40, etc. And the surface markers for the M2 phenotype are Ym1, CD206, CD68 and Arg1. The abnormal expression of translocator protein (TSPO) is considered a marker of microglia activation, anyway (55). PET imaging studies have found that compared with non-depressed patients, depressed patients have an increased density of TSPO ligands in the PFC, ACC, and hippocampal structures, showing increased uptake of TSPO ligands.

Pathogen-associated molecular patterns (PAMPS) or damage-associated molecular patterns (DAMPS) can stimulate resting-state microglia through Toll-like receptors (TLRs) or ATP receptors, respectively, and convert them to the M1 type under the action of IFN-γ (56). M1 microglia stimulation can lead to inflammatory cascades and neuronal degeneration by secretion of pro-inflammatory cytokines such as TNF-α, IL, IFN, NO, ROS, etc. Anti-inflammatory transmitters, such as IL-4 and IL-3, can induce the transformation of microglia to the M2 phenotype (57). M2 glial cells, on the other hand, inhibit inflammation and provide neuroprotection through the secretion of transforming growth factor-β (TGF-β), IL-10, and neurotrophic factors. M1 and M2 regulate the development and regression of neuroinflammation in MDD. For instance, exercise was shown to improve MDD-like behavior in CUMS mice by restoring this balance via the Lipocalin/AdipoR1 pathway (33). Since the development of MDD is closely related to microglia, it has been suggested that MDD is a “microglia disease” (26). Consistently, this idea has been supported by many studies, including one study by Steiner et al. (58), which showed that suicidal depressive patients had increased microglia density in the dorsolateral prefrontal cortex (dlPFC), anterior cingulate cortex (ACC), and the mid-thalamus compared with a healthy population. In a non-inflammatory rodent model, researchers exposed mice to chronic stress conditions, and found that the mice exhibited depressive-like behaviors with significant activation of microglia in their hippocampal structures (59). These studies have proved that microglia activation is an important process that leads to neuroinflammation and induces MDD.

Interestingly, it is recently found that gut microbiota is a critical regulator of microglial function and stress susceptibility. Some studies reported that germ-free mice exhibited reduced expression of genes associated with inflammation and immune surveillance in microglia, alongside higher cell density and distinct morphological features—including a more segmented morphology, longer processes, and increased branching complexity (60). Conversely, fecal microbiota transplantation from mice subjected to CUMS induced microglial priming in the hippocampal dentate gyrus (DG), characterized by hyper-ramified morphology. It is suggested that gut microbiota modulates microglial development and function through three primary mechanisms (28): (a) Microbial metabolites directly influence microglial maturation and activation states; (b) Immune-mediated regulation: The microbiota indirectly modulates microglial morphology, abundance, and function via cytokines produced by peripheral immune cells; (c) Neural signaling: Vagal nerve stimulation by the microbiota suppresses pro-inflammatory cytokine production (e.g., IL-1β, IL-6) in microglia (28). Thus targeted modulation of gut microbiota or inhibition of microglial activation represents a promising therapeutic strategy. For instance, *Lactobacillus reuteri* has been shown to ameliorate anxiety- and MDD-like behaviors in mice by reducing microglial hyperactivation (61, 62). Beyond *Lactobacillus reuteri*, the administration of beneficial bacterial strains such as Blautia producta, some of *Lactobacillus* and *Bifidobacterium* strains, restores homeostatic microglial function and attenuates neuroinflammatory responses (63). However, inconsistencies exist regarding how specific microbial metabolites directly mediate neuroprotection, highlighting the need for standardized methodologies and longitudinal studies (64).

### 3.2 Astrocytes, microglia and neuroinflammation

Astrocytes are also involved in neuroinflammation in MDD via interaction synergistically with microglia, for example, upon microglial activation, astrocytes can amplify inflammatory signals and produce neurotoxic factors (65–67). An examination of the brains of deceased individuals who had experienced severe MDD showed a reduction in both the quantity and concentration of astrocytes in their brain tissue (68). In animal models of MDD, astrocyte activation and increased expression of IL-1β, TNF-α, and IFN-γ can be found in the brains of MDD animals (69). Mice exposed to chronic unpredictable mild stress (CUMS) were shown to have increased GFAP expression in brain regions associated with depressive behaviors, and this was accompanied by the destruction of gut microbiota (altered ratio of Lactobacillus to Clostridium (70). In addition, repetitive transcranial magnetic stimulation (rTMS) and fluoxetine treatment was effective in alleviating depression-like behaviors by weakening the activity of astrocytes (71). Similarly, sodium butyrate therapy significantly increases the abundance of beneficial bacteria, such as *Christensenellaceae*, *Blautia*, and *Lactobacillus* (72).

Astrocytes are the most sensitive cells in the brain, that can sense the environmental changes such as cytokines, metabolites (73), and astrocytes respond to local signals within the brain but are also modulated by the gut microbiota. As an environmental factor, the gut microbiota can directly or indirectly influences astrocyte development, maturation, and functionality via changing intestinal permeability to increase/reduce inflammatory spread, and thus astrocyte hyperactivation (74). This regulation by increasing/decreasing gut permeability, limiting the entry of toxic substances into the bloodstream, thereby changing inflammation spread and astrocyte overactivation, leading to central neuro-inflammatory effects, and thus MDD and anxiety-like behavior in mice (75).

Broadly, gut microbiota can regulate astrocyte through immune, vagal, neuroendocrine, and microbial metabolite-mediated pathways. Immune pathways play a pivotal role in preserving the homeostasis between the gut microbiota and astrocytes. Gut dysbiosis generates pro-inflammatory cytokines that cross the BBB, activating astrocytes and microgila to drive their transition to an inflammatory phenotype. Under steady-state conditions, TRAIL (TNF-related apoptosis-inducing ligand) expression in astrocytes is driven by interferon-γ (IFNγ) produced by meningeal natural killer (NK) cells. LAMP1^+^/TRAIL^+^ astrocytes restrict neuroinflammation by inducing T-cell apoptosis. In contrast, vagal signaling mediates microbiota-astrocyte crosstalk (76), by stimulation anti-inflammatory cytokines to suppress neuroinflammation, and results in therapeutic potential for treatment-resistant MDD (77, 78). Selective serotonin reuptake inhibitors (SSRIs) can enhance vagal nerve activity, thereby increasing sleep and astrocyte reactivity. Paroxetine, a selective serotonin reuptake inhibitor (SSRI), suppresses neuroinflammation through inhibition of the nuclear transcription factor-κβ (NF-κβ) signaling pathway. Importantly, subdiaphragmatic vagotomy has been demonstrated to abolish the antidepressant effects of SSRIs, indicating that vagus nerve-mediated communication between the gut microbiota and astrocytes is essential for their therapeutic efficacy. In addition, gut microbial dysbiosis increases BBB permeability, allowing metabolites such as SCFAs and LPS to traverse the BBB and activate astrocytes (56).

### 3.3 Microglia in neuroinflammation in MDD

**Figure 2:**
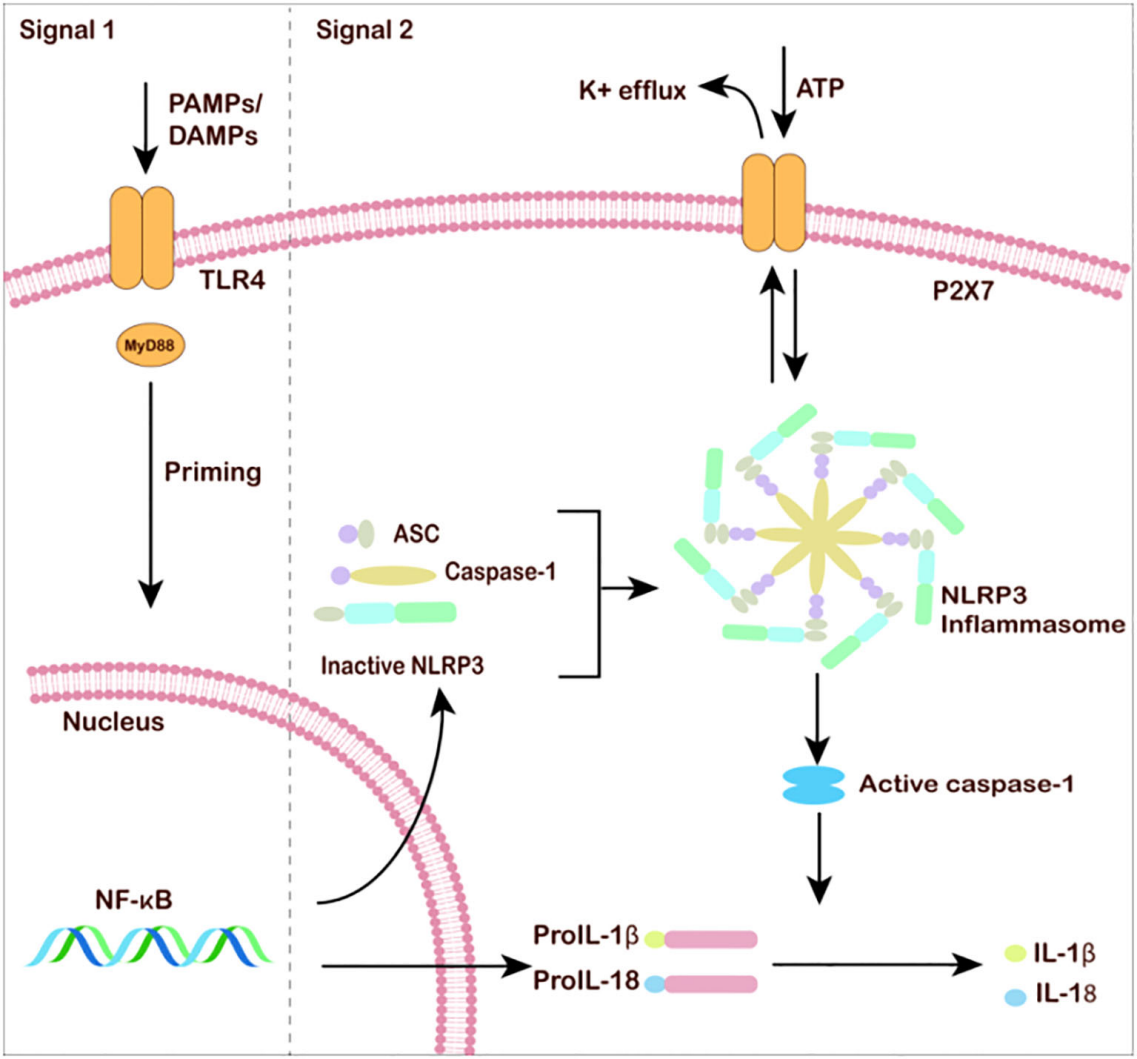
Microglia and neuroinflammation under MDD. The classic NLRP3 inflammasome activation requires two steps: the priming process involves pathogen-associated molecular patterns (PAMPs)/damage-associated molecular patterns (DAMPs) or cytokine-induced NF-κB activation. After NF-κB enters the cell nucleus, the transcription of inflammasome-related genes is unregulated. The activation process requires various exogenous and endogenous molecules (such as ATP, etc.). Other signaling events, such as K^+^ efflux, reactive oxygen species (ROS) generation, and mitochondrial dysfunction, promote NLRP3 inflammasome assembly. Caspase-1 self-activates and cleaves pro-IL-1β and pro-IL-18. Mature IL-1β and IL-18 are released into the extracellular space to amplify the inflammatory response.

There are two steps that activate inflammation in microglia: Toll-like receptors (TLRs) or ATP receptors. In response to chronic stress, PAMPs or DAMPs activate pattern-recognition, neuroinflammation in microglia can be activated by damage-associated molecular pattern (DAMP) or pathogen- associated molecular pattern (PAMP) to activate NFκB, and TLR-mediated activation of the NF-κβ pathway serves as the first signal and promotes the up-regulation of pro-IL-1β and pro-IL-18. As a second step, ATP activation of P2X7R encourages the binding of NLRP3 to apoptosis-associated speck-like protein containing a CARD (ASC) and activates caspase-1, which converts and releases IL-1β and IL-18 precursors into mature forms (**Figure 2**). The activation process of IL-1β and IL-18 precursors requires various exogenous and endogenous molecules (such as ATP, etc.) and other signaling events, such as K^+^ efflux, reactive oxygen species (ROS) generation, and mitochondrial dysfunction, which promote NLRP3 inflammasome assembly. Caspase-1 can self-activate and cleaves pro-IL-1β and pro-IL-18 into mature forms IL-1β and IL-18, which are released into the extracellular space to amplify the inflammatory response. Many current studies focus on the ATP-P2X7R/NLRP3 signaling pathway to study the process of microglial cell inflammatory in MDD.

### 3.4 Molecular mechanism of neuroinflammation in MDD

#### 3.4.1 TLRs, NF-κB, and MDD

TLRs belong to a family of pattern recognition receptors, the first line of immune defense in the human body. Even though there are a diverse repertoire of TLRs (e.g., TLR2 for bacterial lipoproteins, TLR3 for dsRNA, TLR7/8 for ssRNA, TLR9 for CpG DNA), TLR4 is arguably the most critical member in the context of sensing specific danger signals associated with neuroinflammation macrophages and microglia express. TLR4 recognizes PAMPs and DAMPs, as well as heat shock proteins, high mobility group proteins, LPS, and microbial-associated pattern molecules in macrophages and microglia. It is believed that TLR4 receptors can signal through both MyD88 and non-MyD88 pathways to activate NF-κB, thereby promoting the production and release of pro-inflammatory cytokines and chemokines such as IL-1β and TNF-α.

There is an increasing body of evidence suggesting that TLR4 activation plays a vital role in neuroinflammation and is an independent risk factor for MDD severity. Some studies found that experimental animals given lipopolysaccharide (LPS), an agonist of the TLR4 receptor, can exhibit depressive-like behaviors such as decreased interest, pleasure, appetite, and increased despair time (79). Elevated levels of TLR4 expression can be found in the hippocampus in chronic stress animal model, and TLR4 signaling is responsible for sex differences in persistent depressive behaviors in mice (8). Many studies showed a positive correlation between depressive-like behavior and TLR4 levels, and similar studies found that the expression levels of TLR-4 and NF-κB in peripheral blood mononuclear cells of depressed patients were higher than those of non-depressed patients (73), and the previously higher TLR4 mRNA levels were decreased after antidepressant treatment (80).

#### 3.4.2 ATP/P2X7R and MDD

Stress induced ATP release plays a pivot role in neuroinflammation in MDD patients, and P2X7 receptor (P2X7R), an ATP-gated non-selective cation channel, that is widely expressed in glial cells and neurons, is involved in the regulation of cell proliferation and apoptosis, sensory pathways, and immune response. P2X7R not only promotes microglia migration and phagocytosis but also regulates microglia secretion of pro-inflammatory factors and chemokines. Studies have shown that extracellular ATP can combine with P2X7R to promote the release of pro-inflammatory factors, such as IL-1β and IL-18, which enhance neuroinflammation (81). P2X7R is also expressed in astrocytes and is involved in a variety of their physiological functions, such as glutamate release and glutamate excitotoxicity inflammation (82).

Several studies have also confirmed the involvement of P2X7R in the development of neuroinflammation which triggers MDD, and it is found that the expression of P2X7 is positively correlated with MDD. For example, Ren et al. (83) have reported that injection of the P2X7R agonist, adenosine 5’-triphosphate, or its structural analog, dibenzoyl-ATP, aggravated depressive-like behavior in mice. In an acute restraint stress model, rats showed increased inflammation by increasing large amounts of ATP and activating P2X7R (84). Xie et al. (85) demonstrated that the P2X7R receptor antagonist, Kaumas Brilliant Blue G (BBG), down-regulated the expression of P2X7 and NLRP3, limiting pro-inflammatory factors and helping to alleviate the central inflammatory environment. P2X7R knockout mice no longer showed depressive or anxious behaviors after exposure to chronic psychological stress (86). Li (Li et al. (87) found that Na^+^/K^+^-ATPase α1 (NKAα1) can form a complex with the P2X7R receptor under pathological conditions to promote the activation of microglial cell inflammation, and applying a monoclonal antibody to stabilize the membrane NKAα1 to block the inflammation can alleviate MDD symptoms, suggesting a new therapy target.

#### 3.4.3 NLRP3/Caspase-1 and MDD

The relationship between the NLRPs (NOD-, LRR, and pyrin domain containing) family and MDD has attracted much attention in recent studies, particularly the NLR family pyrin structural domain 3 (NLRP3). NLRP3 is activated enzymatically and binds to apoptosis-associated speck-like protein (ASC), which recruits pro-Caspase1 protein, to form NLRP3 inflammatory vesicles. NLRP3 inflammatory vesicles mediate the proteolytic cleavage of pro-Caspase1, activating caspase-1 and ultimately inducing the release of pro-inflammatory cytokines. The activity of NLRP3 inflammatory vesicles and its moderate regulation determine the morphology of microglia and the intensity of neuroinflammatory responses (**Figure 2**) (88).

It has been shown that patients diagnosed with MDD have significantly elevated mRNA levels of NLRP3 and caspase-1 in their blood (89). Knockout of either NLRP3 or caspase attenuates depressive-like behaviors in mice after chronic stress (90, 91). Under physiological conditions, IL-1β induced by NLRP3 inflammatory vesicles is essential for emotional responses and learning (92). However, high levels of IL-1β induce abnormal structural functioning of synapses, which can lead to MDD (93). IL-18 is considered a predictor of MDD risk, and Wu et al. (46) found that IL-18 injected into the amygdala increased depression-like behavior in mice. The inhibitor MCC950 reduced NLRP3, IL-1β, and IL-18 levels in the hippocampus and improved depression behavior (94). These studies suggest that NLRP3 inflammatory vesicles and downstream signaling pathways are essential in neuroinflammation and MDD.

### 3.5 Gut microbiota modulates the inflammation pathways

Gut microbiota collaborates with the molecular mechanisms described above to regulate neuroinflammation, forming an intricate gut-brain axis network. For example, LPS and butyrate are two important microbial metabolites in neuroinflammation. Butyrate produced by the Lachnospiraceae family inhibits histone deacetylase (HDAC), thereby blocking the nuclear translocation of the NF-κB p65 subunit. Meanwhile, LPS binds to the TLR4/MD2 complex, triggering MyD88/TRIF-dependent IKK phosphorylation, thereby releasing IκB’s inhibition of NF-κB and driving the transcription of TNF-α and IL-6. And improving LPS levels and pro-inflammatory cytokines (IL-1β, IL-6, TNF-α) and inhibiting the TLR4/MyD88 pathway with herbal formula Zuogui Jiangtang Jieyu (ZJJ) by downregulating Gram-negative bacteria abundance alleviated diabetes-associated MDD (95). Another example, chicoric acid (CA) attenuated neuroinflammation by suppressing the TLR4/MyD88 pathway and restoring gut microbiota balance (96). In addition, Histicola and Bifico have been shown to increase the abundance of gut microbiota (Lactobacillus, Desulfovibrio, Akkermansia), downregulate LTR4/NF-κB, and improve estrogen-induced MDD (97, 98).

P2X7R is expressed in intestinal cells and plays a role in both health and disease in the gastrointestinal system. Under pathological conditions, the intestinal barrier is disrupted, leading to decreased levels of ZO-1 and claudin-1, increased ATP release by the microbiota, and activation of P2X7R. When the concentration of extracellular ATP produced by the intestinal microbiota is high, the sIgA response induced by intestinal lymphoid tissue is suppressed, thereby facilitating the colonization of pathogenic bacteria (99). However, BBG(P2X7R antagonist) reversed the relative abundance of Bacteroidetes and Akkermansia in alcohol-fed mice. alcohol-fed mice (100).

The assembly and activation of NLRP3 inflammasomes are key steps in the process by which gut microbiota dysbiosis drives neuroinflammation, which in turn promotes the onset and progression of MDD. Caspase-1 inhibition via genetic knockout or pharmacological intervention reduces depression and anxiety-like behaviors in mice. Depressive behavior was improved in NLRP3-/- mice, and the types of gut bacteria (Firmicutes, Proteobacteria, Ruminococcus, Prevotella, Bacteroidetes) were also altered (101). Under steady-state conditions, SCFAs exert a dual regulatory effect on NLRP3, primarily exerting anti-inflammatory effects. Studies have shown that under TLR-activated conditions, SCFAs can shift from anti-inflammatory to pro-inflammatory effects by inhibiting HDAC activity, activating the NLRP3 inflammasome, and promoting the release of IL-1β (102). Fecal microbiota transplantation (FMT) from healthy mice to postpartum MDD (PPD) mice alleviated depression/anxiety-like behaviors, reduced NLRP3/caspase-1-mediated inflammation in the gut and hippocampus, increased SCFA levels. In contrast, enriching beneficial bacteria (e.g.,Lactobacillus) restored gut dysbiosis and reducing Akkermansia abundance (103), and exercise intervention increased Lactobacillus abundance while suppressing NLRP3/caspase-1 pathway activation (104).

## 4 The effects of neuroinflammation on the nervous system

### 4.1 Neuroinflammation affects synaptic plasticity

Synaptic plasticity underpins both structural and functional plasticity of the nervous system, with structural plasticity denoting alterations in the quantity, morphology, and architecture of the pre- and post-synaptic membranes and their interstices resulting from internal and external stimuli. Conversely, functional plasticity pertains to alterations in the intensity or efficacy of synaptic transmission, primarily long-term synaptic plasticity, encompassing long-term potentiation (LTP) and long-term MDD (LTD). Impaired synaptic plasticity can readily result in cognitive and emotional learning and adaptions, and finally emotional related problems, including MDD.

In the resting state, microglia govern synaptic pruning to enhance synaptic functional stabilization. When glial cells are activated due to neuroinflammation, too much secreted pro-inflammatory cytokines influence synaptic plasticity. Nguyen et al. (105) have shown that IL-33 signaling drives experience-dependent synaptic plasticity in the hippocampus with IL-33 receptor depletion from microglia, resulting in decreased dendritic spines and newborn neurons. In addition, the concentration of tumor necrosis factor α (TNF-α) varies in terms of its regulation of synaptic plasticity, with low concentrations of TNF-α promoting synaptic plasticity and high concentrations, particularly under inflammatory conditions, inducing dysregulation of synaptic transmission and plasticity. TNF-α production also encourages the release of glutamate from astrocytes, which enhances excitotoxicity in neurons (106). Thus, it is clear that inflammation is extensively involved in neuronal damage, which is closely related to MDD.

### 4.2 Neuroinflammation inhibits hippocampal neurogenesis

Chronic stress can lead to atrophy, apoptosis, and reduction in the number of hippocampal neurons; thus, neuronal regeneration is a crucial mechanism for recovery from MDD (2). Neurogenesis, the mechanism through which neural stem cells (NSCs) generate new neurons, enhances the brain’s structural plasticity. While some previous views suggested that this phenomenon is limited to childhood or early adolescence, some recent studies (107) reveal that neurogenesis continues throughout adulthood, for example, the hippocampus, situated in the medial temporal lobe, is a vital anatomical area within the limbic nervous system. It governs advanced neural functions, including mood and cognition, and is one of the brain regions recognized for neurogenesis. It is suggested that adult neurogenesis is limited to the subventricular zone (SVZ) of the lateral ventricles and the subgranular zone (SGZ) of the dentate gyrus (DG) of the hippocampus under physiological conditions. However the neurogenesis might involve all hippocampal regions under pathological condition, and this study has garnered significant focus in contemporary MDD research, and has evolved into a new field of study, namely adult hippocampal neurogenesis (AHN),.

Adult hippocampal neurogenesis (AHN) refers to the neuronal proliferation, differentiation, and maturation processes in the adult hippocampus. The AHN comprises a sequence of neurogenic cascade reactions wherein the DG undergoes asymmetric divisions to produce neural progenitor cells (NPCs), which subsequently differentiate into adult neuronal cells. The surviving adult neuronal cells evolve into immature neurons, ultimately maturing into DG granule neurons that integrate into the established hippocampal circuitry. The heightened expression of pro-inflammatory genes in mice subjected to Repeated social defeat (RSD) indicated a notable decrease in early neuronal markers DCX on day ten post-RSD. At the same time, impaired differentiation of NPC neurons proliferating during RSD was observed after 28 days, coinciding with the activation of neuroinflammation (108). Administration of anti-inflammatory medications, such as NSAIDs, cytokine inhibitors, statins, and minocycline, has been demonstrated to reduce the hippocampus neurogenesis (109–111). Neuroinflammation diminishes hippocampus neurogenesis, thereby leading to depression-like behaviors.

Indeed, it is found that the number of NCSs in the DG region of the hippocampus is reduced in MDD patients, and vascularization is decreased. In addition, neuroimaging studies have shown (112) that hippocampal volume is reduced in the brains of depressed patients compared with healthy persons. Studies in animals demonstrated that prolonged chronic stress stimulates the proliferation of hippocampal neurons. At the same time, antidepressant administration enhances the quantity of hippocampus NPCs and the expression of trophic factors, among other effects (113). Zhang et al. (114) reported that chronic stress diminishes the expression of Nuclear Receptor Binding Factor 2 (NRBF2), leading to the disruption of neurogenesis-related protein networks, a reduction of NCSs and that the overexpression of NRBF2 mitigates MDD-like behavior induced by CSDS.

### 4.3 Neurotrophic factors

Briain derived neurotropic factor (BDNF) is one of the most well-studied neurotrophic factors, a key player in psychiatric disorders, and one of the reliable biomarkers used to monitor MDD. BDNF can initiate multiple signaling pathways by activating distinct receptor types, primarily the TrkB and the p75 neurotrophic factor receptor (p75NTR) pathways, which can activate phosphatidylinositol 3-kinase (PI3K)-AKT and extracellular signal-regulated kinase (ERK) by binding to TrkB, etc (115), and chronic exposure to stress or inflammation leads to decreased BDNF levels (116), which can also be found in animal models of MDD.

Cytokines released during glial cell activation at neuroinflammation are posited to obstruct neurotrophic signaling in neurons, thereby impeding BDNF-induced activation of PI3K and Erk1/2, which subsequently hinder axon growth and neuronal marker expression, culminating in neuronal apoptosis (117). For example, the flavonoid leucovorin has been shown to reduce pro-inflammatory cytokine levels (118) and has recently been shown to enhance the TrkB/BDNF pathway, and stimulate FGF/FGFR1 signaling, which helps up-regulate the expression of BDNF, promote the differentiation of NCSs and neuronal growth, and also provide a new therapeutic strategy for MDD (119). Other neural growth factors (NGF) have also been demonstrated to influence neuroinflammation, with NGF blocking TLR4-mediated activation of the NF-κB and JNK pathways and attenuating pro-inflammatory responses in glial cells (120). NGF has been known to accelerate macrophage polarization of the M2 phenotype and to increase secretion of pro-regenerative factors (GAP-43, NF-200), thereby facilitating neuroinflammatory responses (121).

## 5 Conclusion and limitations

With the advances of research on MDD, there has been a better understanding of the specific mechanisms and signaling pathways involved in MDD. The brain-gut axis, which is a complex interactive system, plays a significant role in the pathophysiology of MDD. Dysbiosis of the gut microbiota can produce metabolites that compromise barrier functions and trigger the release of inflammatory factors. Additionally, these metabolites activate glial cells, leading to neuronal damage, synaptic dysfunction, and inhibition of hippocampal neurogenesis, ultimately contributing to neuroinflammation. Therefore, targeting the brain-gut axis to suppress glial cell activity and reduce their inflammatory activation may represent a potential therapeutic strategy for MDD. In addition, sleep disorders, which is related with both the etiology of MDD and also affects the gut microbiota, can modulate the inter-play among the gut microbiota and neuroinflammation.

However, many concerns about precise mechanisms of the brain-gut axis in MDD remain to be elucidated. Further elucidation of these mechanisms will lay the foundation for developing novel antidepressant drugs, breaking through the limitations of traditional monoamine-based treatments. For example, traditional monoamine hypothesis suggested that MDD is due to limitation of the three monoamines (dopamine, norepinephrine and 5-HT), and antidepressants that can increase in these three monoamines can alleviate MDD. The role of brain-gut axis in modulation of the monoamines are still need to be explained, indeed, 90–95% of serotonin in the whole body is produced in the gastrointestinal tract, thus the interplay between brain-gut axis with monoamines might shed more light on the mechanisms of MDD.
